# Effects of word predictability on eye movements during Arabic reading

**DOI:** 10.3758/s13414-021-02375-1

**Published:** 2021-10-10

**Authors:** Maryam A. AlJassmi, Kayleigh L. Warrington, Victoria A. McGowan, Sarah J. White, Kevin B. Paterson

**Affiliations:** 1 Department of Psychology, Zayed University, Dubai, UAE; 2 Department of Neuroscience, Psychology and Behaviour, University of Leicester, Leicester, UK; 3 Department of Psychology, Nottingham Trent University, Trent, UK

**Keywords:** Arabic, Eye movements during reading, Word predictability, Word-skipping

## Abstract

Contextual predictability influences both the probability and duration of eye fixations on words when reading Latinate alphabetic scripts like English and German. However, it is unknown whether word predictability influences eye movements in reading similarly for Semitic languages like Arabic, which are alphabetic languages with very different visual and linguistic characteristics. Such knowledge is nevertheless important for establishing the generality of mechanisms of eye-movement control across different alphabetic writing systems. Accordingly, we investigated word predictability effects in Arabic in two eye-movement experiments. Both produced shorter fixation times for words with high compared to low predictability, consistent with previous findings. Predictability did not influence skipping probabilities for (four- to eight-letter) words of varying length and morphological complexity (Experiment 1). However, it did for short (three- to four-letter) words with simpler structures (Experiment 2). We suggest that word-skipping is reduced, and affected less by contextual predictability, in Arabic compared to Latinate alphabetic reading, because of specific orthographic and morphological characteristics of the Arabic script.

## Introduction

During reading, the eyes move in a series of rapid, ballistic movements (saccades) that carry the reader’s gaze across lines of text, separated by brief fixational pauses during which individual words are processed in high acuity (Liversedge & Findlay, 2000; Rayner, 1998, 2009). Substantial research has focused on understanding this eye-movement behaviour and, in particular, whether it is under cognitive control and so influenced by aspects of online language processing, such as lexical access (see, e.g., Rayner et al., 1996).

Findings from numerous studies suggest that forward-directed eye movements in reading are influenced by a combination of low-level visual information about the length and location of words, and higher-level knowledge about a word’s frequency of written usage and its predictability from the prior sentence context (for reviews, see Rayner, 1998, 2009). These factors have been shown to influence the probability of a word being fixated (and therefore whether it is skipped), as well as how long the eyes dwell on fixated words. Specifically, studies show that words that are shorter, of higher lexical frequency, or more contextually predictable have lower fixation probabilities (and so are skipped more often). Moreover, when fixated, these words have shorter fixation times (e.g., Altarriba et al., 1996; Choi et al., 2017; Gollan et al., 2011; Hand et al., 2010; Inhoff & Rayner, 1986; Joseph et al., 2009; Kliegl et al., 2004; Paterson et al., 2013a, 2013b; Rayner, 1979; Rayner et al., 1996, 2011; Rayner et al., 2004; Rayner & McConkie, 1976; Staub & Benatar, 2013). Such findings are important as they demonstrate that decisions about when and where to move the eyes in reading are influenced by factors affecting the process of word identification. Moreover, such research has been fundamental to the development of sophisticated computational models of eye-movement control in reading, including the E-Z Reader (e.g., Reichle et al., 1998) and SWIFT (e.g., Engbert et al., 2005) models (for a recent review, see Reichle, 2021).

While both types of models can accurately simulate forward-directed eye movements in reading, there are crucial differences in their underlying assumptions. One approach, exemplified by the E-Z Reader model, assumes that word identification is the engine that drives the forward movement of the eyes. Within this model, attention is allocated to words strictly serially, so that they are identified one at a time. In an initial stage of word identification, called the *familiarity check* (or L1), the system computes whether a word is likely to be identified imminently, which triggers the programming of a saccade (i.e., eye movement). In a second stage (L2), lexical access is completed. Both stages are assumed to be influenced by lexical variables, including word length, frequency and predictability. In the case of LI, these variables are thought to influence the probability and duration of fixations on words. In particular, it is assumed that the time taken to complete the familiarity check for the currently fixated word (word *n*) is a function of these variables. Once this is completed, covert attention is assumed to shift from this word to the following word (word *n*+1), initiating a new familiarity check for *n*+1 and the programming of a saccade to that word. Crucially, if the *n*+1 familiarity check can be completed before the saccade program is ready, the system may revoke this saccade program and initiate a new one that skips *n*+1 and targets a saccade towards the next word along.

By comparison with E-Z Reader, SWIFT is based on the assumption that multiple words are processed in parallel within a region around each fixation location. Within this model, it is assumed that saccade timing is primarily regulated by an autonomous timer that maintains a preferred reading speed, and that saccades are targeted towards words based on their patterns of lexical activation. As with E-Z Reader, this process can be influenced by lexical variables, including word length, frequency and predictability. However, word frequency and predictability have a lesser role in the SWIFT model and are assumed to influence oculomotor processes only occasionally. For instance, the initiation of a saccade may be interrupted, so that the reader dwells on a word for longer if this word proves difficult to identify because of its low lexical frequency or low predictability. Similarly, while word-skipping is assumed to be driven primarily by word length, knowledge about a word’s frequency and predictability can influence the selection of saccade targets, producing increased skipping rates for words that are of high lexical frequency or highly predictable. Accordingly, despite differing in their emphasis on cognitive factors, both models allow for factors affecting the identification of words to play a role in eye guidance.

These influences on eye guidance have been extensively investigated in writing systems based on the Latin alphabet, such as English and German. However, comparatively little research has investigated effects in Semitic scripts such as Arabic and Hebrew, despite these having very different visual and linguistic characteristics (see, e.g., AlJassmi et al., 2021). Such knowledge is nevertheless crucial for establishing the generality of mechanisms of eye guidance across different alphabetic writing systems. Of these languages, Hebrew has received most attention to date, although eye-movement studies in this script largely focus on morphological and syntactic processing (e.g., Deutsch, 1998; Deutsch et al., 2000; Deutsch et al., 2003; Deutsch et al., 2005; Deutsch et al., 2018; Deutsch et al., 2021; Deutsch & Bentin, 2001; Kuperman & Deutsch, 2020), with few studies investigating lexical influences on eye movements. A key study, by Deutsch and Rayner (1999), showed that word length has a similar influence on eye movements in Hebrew reading as observed for Latin-based scripts like English, despite its different reading direction. Currently, there are few eye-movement studies in Arabic, although several recent studies have investigated effects of word length and frequency (Hermena et al., 2017; Hermena et al., 2019; Paterson et al., 2015), showing that these variables can influence fixation times for words but have only small or non-significant influences on word-skipping. Accordingly, the present research aimed to extend our understanding of lexical influences on eye guidance in Arabic reading by examining word-predictability effects.

As noted above, research with Latin-based languages suggests that, when reading a sentence beginning “The manager tends to be in a very bad mood every morning before he has his…”, whether the next word in the sentence is highly predictable (e.g., “coffee”) or less predictable (e.g., “drink”) can affect eye movements. In particular, even though both words are possible continuations, the more predictable word will have a higher skipping probability and receive shorter fixations. Our goal was to establish whether similar predictability effects are observed in Arabic reading. While we might expect to observe such effects in fixation times on words, there were reasons to doubt whether predictability would influence word-skipping. It was of particular concern that words are skipped rarely in Arabic reading. Previous Arabic studies report low word-skipping rates (< 10% prevalence; Hermena et al., 2017, Hermena et al., 2019; Paterson et al., 2015), as compared to 20–30% prevalence for skilled readers of Latin-based languages (Brysbaert et al., 2005; Rayner, 1998, Rayner, 2009). This high rate of word-skipping for Latin-based scripts is partially attributable to the frequent skipping of short, predictable words in these scripts (see Brysbaert et al., 2005; Rayner & McConkie, 1976; Rayner et al., 1996). While Arabic words are generally short, with four- to eight-letter words accounting for 90% of items in the Aralex word database (Boudelaa & Marslen-Wilson, 2010), this is because vowels are omitted in most forms of Arabic text unless required to disambiguate a word (with the exception of religious texts and texts designed for beginning readers). Moreover, although words tend to include multiple affixes (prefixes, suffixes and infixes) to convey grammatical information, these are represented by few letters. Importantly, however, predictability has been shown to influence fixation probability and duration for both short and long words in scripts like English (Rayner et al., 2011), raising the question of whether similar effects might be observed for Arabic.

The decision to skip a predictable word is necessarily based on both information obtained from outside of foveal vision (i.e., in parafoveal vision) and predictions derived from the prior sentence context. Such decisions will therefore depend on the strength of the predictions and the quality of parafoveal information, including available information about word length and orthography (e.g., Balota et al., 1985; Chang, Hao, et al., 2020a; Chang, Zhang, et al., 2020b; Choi et al., 2017; Juhasz et al., 2008; Schotter et al., 2015; Staub, 2020; Staub & Goddard, 2019; Veldre & Andrews, 2018; White et al., 2005). One possibility is that the available parafoveal information does not support predictability effects on word-skipping in Arabic. There are several reasons this might be the case. A first is that Arabic uses a semi-cursive script in which letters are connected by ligatures (small lines), while the orthography includes letters of varying width and groups of letters that are differentiated by only minor visual differences (e.g., ,  and , differ only by the presence and location of small dots, while  and  differ slightly in shape and number of small dots). The semi-cursive nature of the script and variation in letter width may create a situation in which a word’s physical length does not help constrain word identities, as words with the same number of letters can differ in physical length, while words with different numbers of letters can have the same physical length (Hermena et al., 2017). A further possibility is that similarity in the shape of groups of letters may impair the discriminability of letters in parafoveal words. Studies using masked priming techniques suggest that the influence of this letter similarity is relatively small when words are in foveal vision (Perea et al., 2016). However, other research using lateralized displays suggests greater confusability of letter identities outside of foveal vision (e.g., Eviatar et al., 2004). Eviatar et al. further argued that this difficulty is compounded by reading direction. Specifically, they argue that, because Arabic is read from right to left, parafoveal words will be perceived in the left visual field during reading. Information about these words will therefore first project to a reader’s right cerebral hemisphere (because of contralateral retinal projections, e.g., Jordan & Paterson, 2009), which for most (i.e., right-handed) readers has less efficient word-recognition capabilities. Following this account, orthography and reading direction may conspire to limit parafoveal processing in Arabic reading.

Secondly, whereas words in Latinate languages often have informative word beginnings that can help constrain parafoveal word identities (e.g., Farid & Grainger, 1996; Hand et al., 2012; Pagán et al., 2016), Arabic (like Hebrew) uses a non-concatenative morphology in which triples of letters that express a word’s core meaning intermingle with other letters to convey its inflectional meaning. For instance, the root consonants  (often transcribed as “ktb”) express the general meaning of “writing” in Arabic and these consonants intermingle with other letters (as indicated using underlining in the following examples) to create specific meanings, including  (“he writes”)  (“writer”), and  (“writing”). Consequently, letters that express a word’s core meaning are not always contiguous with each other, and can be distributed throughout a word rather than located towards its beginning (Boudelaa, 2013; Ratcliffe, 2013). Readers therefore might not benefit from a parafoveal preview of the beginning letters in Arabic words, as compared to in scripts like English (e.g., Hand et al., 2012; Pagán et al., 2016), as the most informative elements seldom appear at the word beginning (Farid & Grainger, 1996). Finally, because of the script’s agglutinative (or fusional) nature, Arabic words can be highly morphologically complex, using multiple affixes (i.e., prefixes, suffixes and infixes) to convey grammatical information. Words can therefore be informationally dense, making them more difficult to identify parafoveally. This morphological complexity may also incur a foveal processing cost for fixated words that reduces resources available for parafoveal processing (e.g., Henderson & Ferreira, 1990; Payne et al., 2016; Roman & Pavard, 1987).

Given this potential for impoverishment of parafoveal processing, it will be important to establish the influence of word predictability when reading Arabic. This may yield similar effects to Latinate scripts, such that fixation times are shorter and skipping rates higher for more predictable words. Alternatively, effects may be more limited, such that predictability influences reading times but not word-skipping. We conducted two experiments to investigate this. In both, we recorded the eye movements of fluent native Arabic readers while reading sentences containing a target word with high or low predictability from the prior sentence context. Experiment 1 used four- to eight-letter target words varying in morphological complexity, allowing us to assess predictability effects on reading times and word-skipping for typical Arabic words. With Experiment 2, we intentionally selected only three- to four-letter morphologically simple words to test effects under conditions that might promote parafoveal processing and maximise the likelihood of observing a skipping effect.

## General method

### Ethics statement

Ethical approval was received from ethics committees at the University of Leicester and Zayed University.

### Participants

**Fig. 1:**
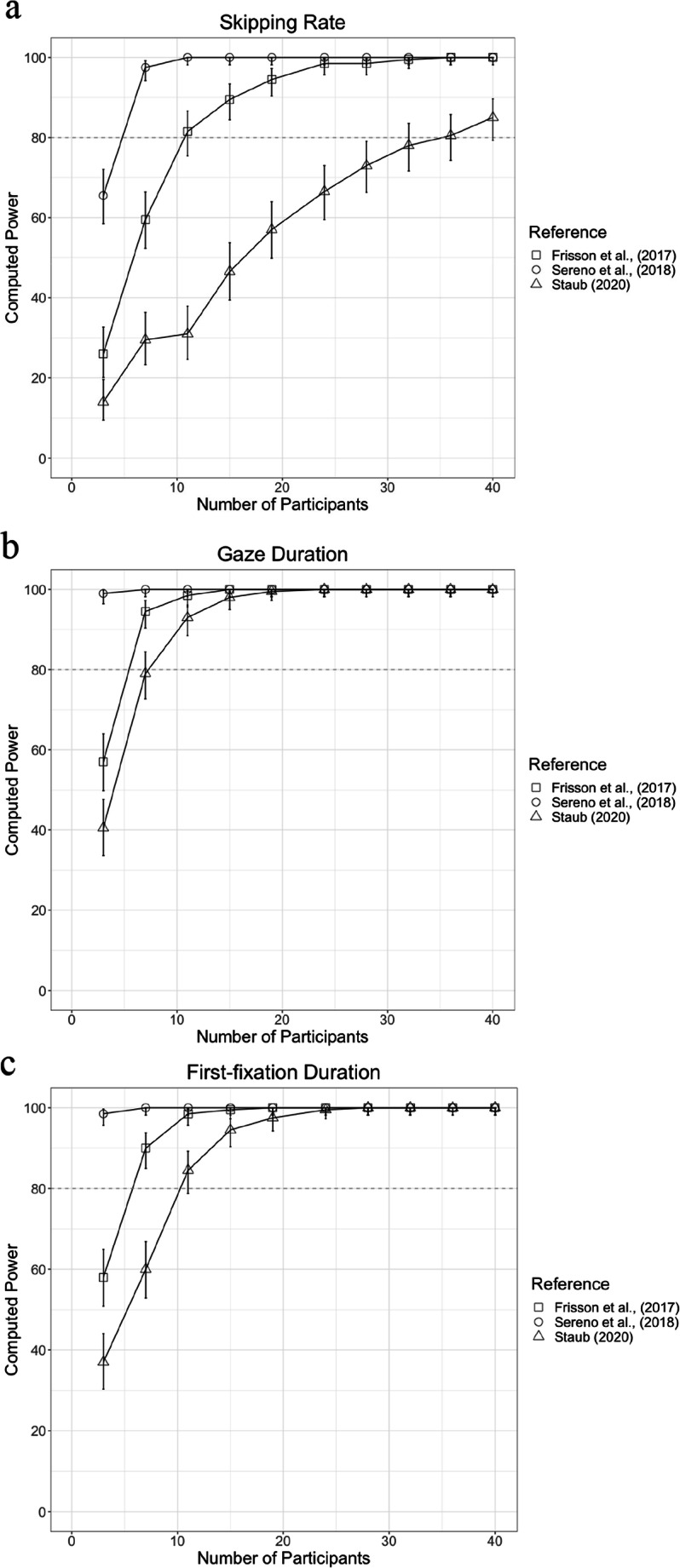
Power Estimates for Experiments 1 and 2 for (**a**) Word-Skipping Probability, (**b**) Gaze Duration for Target Words and (**c**) First-Fixation Duration

In each experiment, 40 fluent native Arabic readers aged 18–28 years (*M* = 21 years; all female) were recruited from Zayed University (13 participated in both experiments). All had normal or corrected-to-normal vision, assessed using a standard eye chart. Three participants from Experiment 1 and one from Experiment 2 were excluded due to poor performance on questions used to test comprehension. To assess the power of our design, we performed a power simulation using the simr package (Green & MacLeod, 2016) in R (R version 4.0.2, R Core Team, 2016). This was conducted for three critical eye-movement variables, word-skipping probability, first-fixation duration, and gaze duration, which are informative about the initial (i.e., first-pass) processing of words. No previous studies have examined word predictability effects on eye movements in Arabic reading. The simulation therefore used effect sizes computed using means and standard deviations from three previous word-predictability studies conducted in English (Frisson et al., 2017; Sereno et al., 2018; Staub, 2020). Power curves based on these effect sizes are shown in Fig. 1. The results indicate that a sample size of 40 participants should be sufficient to detect a word predictability effect of the size reported in previous studies for all three critical variables with at least 80% power (Brysbaert & Stevens, 2018).

### Stimuli and design

**Fig. 2:**
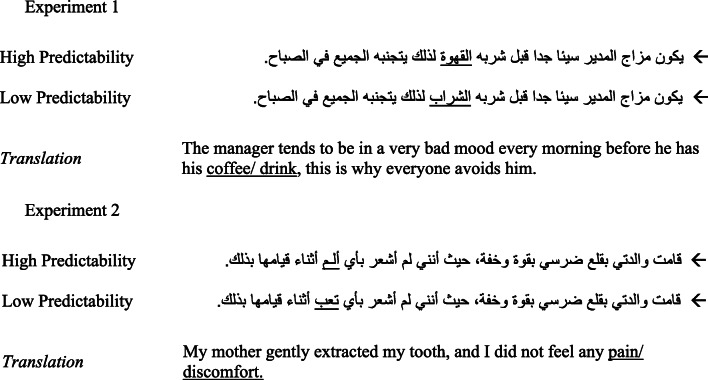
Example sentences in Experiments 1 and 2. Target words are underlined but shown normally in the experiment. Arrows indicate reading direction

Stimuli were two sets of 72 sentence frames that included one of two interchangeable target words (see Fig. 2). These had either high or low predictability from the prior sentence context. Experiment 1 used a variety of four- to eight-letter words and Experiment 2 used three- to four-letter nouns as targets. As Arabic conventionally uses a semi-cursive proportional script, high- and low-predictability words were matched for letter length across high- and low-predictability conditions (Experiment 1, *M* = 6.1, *SD* = 1.1; Experiment 2, *M* = 3.5, *SD* = .5; *t*(143) = -24.48, *p* < .001), and closely for spatial width (Experiment 1, high-predictability = 1.31°, low-predictability =1.29°, *t*(71) = 1.29, *p* = .20; Experiment 2, high-predictability = .89°, low-predictability =1.00°, *t*(71) = 1.23, *p* = .22). The spatial width of words was shorter in Experiment 2 compared to Experiment 1 as a result of selecting words with fewer letters for this experiment (*t*(143) = -14.32, *p* < .001). The high- and low-frequency target words in each experiment were also matched for lexical frequency (in counts per million; Experiment 1, high-predictability = 68.1, *SD* = 152.5, low-predictability = 68.4, *SD* = 190.8, *t*(71) = .01, *p* = .99; Experiment 2, high-predictability = 81.9, *SD* = 84.4, low-predictability = 80.7, *SD* = 169.5, *t*(70) = .01, *p* = .99), using the Aralex database (Boudelaa & Marslen-Wilson, 2010). While target words had numerically higher lexical frequencies in Experiment 2 than in Experiment 1, this difference was not statistically significant (*t*(143) =.68, *p* =.50). Sentences were 10–20 words long and the target word always appeared near the sentence middle. Arabic uses small marks (diacritics) to indicate vowels, primarily in formal texts or to avoid ambiguity in everyday texts (Ratcliffe, 2013). Accordingly, these were added only when words were ambiguous (0.7% of words), and never for target words.

Word predictability was measured using a cloze procedure (Rayner & Well, 1996; Schwanenflugel, 1986; Taylor, 1953). Stimuli for each experiment were truncated immediately prior to the target word and undergraduate participants from Zayed University (24 per experiment), who did not participate in the experiments, provided written completions for each fragment. Target words were considered highly predictable if produced on more than 60% of completions, and of low predictability if produced on less than 15% of completions (Experiment 1, high-predictability, *M* = 89%, *SD* = 10, low-predictability, *M* = 0.4%, *SD* = 2, *t*(71) = 78.52, *p* < .001; Experiment 2, high-predictability, *M* = 90%, *SD* = 9, low-predictability, *M* = 1%, *SD* = 3, *t*(71) = 77.43, *p* < .001). Note that these cut-offs are consistent with cut-offs used in other word predictability studies (Frisson et al., 2017; Miellet et al., 2007; Rayner et al., 2011; Staub, 2020; see Staub, 2015, for a discussion). For each sentence, 24 additional participants (per experiment) rated sentence plausibility on a 5-point scale (1 = highly implausible, 5 = highly plausible). This was high for all sentences, but higher for sentences with highly predictable words than less-predictable words (Experiment 1, high-predictability, *M* = 4.6, *SD* = 0.3, low-predictability, *M* = 3.6, *SD* = 0.6, *t*(71) = 11.78, *p* < .001; Experiment 2, high-predictability, *M* = 4.7, *SD* = 0.2, low-predictability, *M* = 3.4, *SD* = 0.7, *t*(71) = 15.87, *p* < .001), consistent with other research (Frisson et al., 2017).

For each experiment, sentence frame and target word combinations were split into two lists, each containing all 72 frames and an equal number of high- and low-predictability target words. Participants were pseudo-randomly assigned to each list. Sentences in each list were presented in random order to each participant, preceded by eight practice sentences. The experiments had a within-participants design with the factor word predictability (high, low).

### Apparatus and procedure

Eye movements were recorded at Zayed University using an Eyelink 1000 plus (SR Research) tower-mounted eye-tracker. Right eye-gaze location was recorded each millisecond during binocular viewing. This was in line with other research on reading in right-to-left languages, such as Arabic, Hebrew and Uyghur, that have recorded eye movements from the right eye only (Deutsch et al., 2005, 2021; Deutsch & Rayner, 1999; Hermena et al., 2015, 2016, 2017, 2019; Kuperman & Deutsch, 2020; Paterson et al., 2015; Yan et al., 2014; Zhou et al., 2021). Sentences were displayed on a high-definition 24-in. BenQ monitor at 80-cm viewing distance. Text was presented in 14-point using a commonly used proportional font (Arial).

Participants were tested individually. Before the experiment began, participants were instructed to read normally and for comprehension. The eye-tracker was then calibrated using a 3-point horizontal calibration (ensuring spatial error < .30°). Calibration accuracy was assessed before each trial and the eye-tracker re-calibrated as necessary to maintain this low spatial error. At the beginning of each trial, a fixation square equal in size to a character space was presented on the right of the screen (as Arabic is read from right-to-left). Once the participant fixated this location, a sentence was presented with its first letter replacing the square. Once the participant finished reading, they pressed a response button. The sentence then disappeared, replaced on 50% of trials by a comprehension question requiring a yes/nobutton-press response. The experiment lasted about 30 min for each participant.

### Data analysis

Following standard procedures, fixations < 80 ms or > 1,200 ms were discarded (affecting 3.6% fixations in Experiment 1, and 3.2% of fixations in Experiment 2). We also removed trials in which: (1) track loss occurred (affecting < 0.01% of trials in both Experiment 1 and Experiment 2), (2) a blink occurred on the target word or during an immediately adjacent fixation (affecting 11% of trials in Experiment 1 and 6% of trials in Experiment 2), or (3) when saccades to the target word were longer than ten characters (i.e., > 140 pixels, with each character subtending 14 pixels on average; affecting 0.01% of trials in Experiment 1 and 0.02% in Experiment 2). This resulted in the exclusion of 12% of trials in Experiment 1, with 2,542 trials remaining, and 7% of trials in Experiment 2, with 2,671 trials, remaining. Finally, we removed observations more than 2.5 standard deviations from the mean of each participant for each fixation measure (FFD: affecting 2.3% of data in Experiment 1 and 1.7% in Experiment 2; GD: affecting 2.1% of data in Experiment 1 and 1.9% in Experiment 2).

The remaining data were analysed using the lme4 package (version 1.1-27; Bates et al., 2015) in R (R Core Team, 2016). Linear mixed-effects models (LMMs) were used for continuous measures and generalized linear mixed models (GLMMs) for binominal measures. For each measure, a maximal random effects structure was used (Barr et al., 2013), with participants and stimuli as crossed random effects and word predictability as a fixed effect, with all models converging successfully. Contrasts were defined using the contr.sdif function in the MASS package (Venables & Ripley, 2002), with two levels of predictability (high, low). Analyses for untransformed and log-transformed reading times data produced the same patterns of results, so only results for untransformed data are reported for transparency.

Sentence-level analyses were computed to provide normative data for skilled Arabic reading. These comprised reading rate (words per minute), average fixation duration, average number of fixations per word, number of regressions (backward eye movements), and progressive saccade length (average length of forward eye movements, in average character length in the stimulus set). We also report target word-level eye movement measures used to test hypotheses concerning word predictability effects. To decrease the likelihood of Type I error due to testing multiple eye movement variables (von der Malsburg & Angele, 2017), we focused analyses on three first-pass measures that are widely reported in word predictability studies (Balota et al., 1985; Ehrlich & Rayner, 1981; Rayner et al., 2005; Rayner & Well, 1996). First-pass reading refers to processing that takes place during an initial encounter with a word, prior to a fixation on the next words or a regression to re-inspect earlier text. To assess first-pass effects of word predictability, we examined: (1)word-skipping (SKIP), which is the probability of not fixating a word during first-pass reading, (2)first-fixation duration (FFD), which is the length of the first fixation on a word during first-pass reading; and (3) gaze duration (GD), which is the sum of all first-pass fixations on a word. To further reduce the likelihood of a Type I error, we used a Bonferroni correction to adjust for multiple comparisons. To do so, we divided the alpha threshold (0.05) by the number of critical dependent measures (3) to yield an alpha of .02 (*t/z* values = 2.39) for word-level measures of primary interest. We report several additional eye-movement variables as exploratory analyses that include *t/z* values without commenting on their statistical significance. These were single-fixation duration (SFD; length of the fixation on a word receiving only one first-pass fixation), regressions-out (RO; probability of a first-pass regression from a word), total reading time (TRT; sum of all fixations on a word) and regressions-in (RI; probability of a regression back to a word).

## Experiment 1: Results and discussion

Data files and related resources for Experiments 1 and 2 are available from the University of Leicester online Figshare repository: https://figshare.com/s/4382467c9a132fd2e15c

Accuracy for responses to comprehension questions was high (*M* = 96%), above 90% for all participants, indicating good comprehension.

### Sentence-level measures

Table 1 shows mean sentence-level measures. These are similar to those in previous Arabic research (Hermena et al., 2017, 2019; Jordan et al., 2014; Paterson et al., 2015), and so appear typical for Arabic reading.

**Table 1:** Sentence-level measures for Experiments 1 and 2

Measure	Experiment 1	Experiment 2
Reading rate (wpm)	203 (26)	208 (31)
Average fixation duration (ms)	249 (4)	242 (4)
Average number of fixations per word	1.2 (.01)	1.2 (.01)
Number of regressions (%)	13.8 (.9)	13.6 (.9)
Progressive saccade length (chars)	7.3 (.03)	7.2 (.03)

*Note*. The standard error of the mean is shown in parentheses

### Hypothesis-testing analyses for word-level measures

**Fig. 3:**
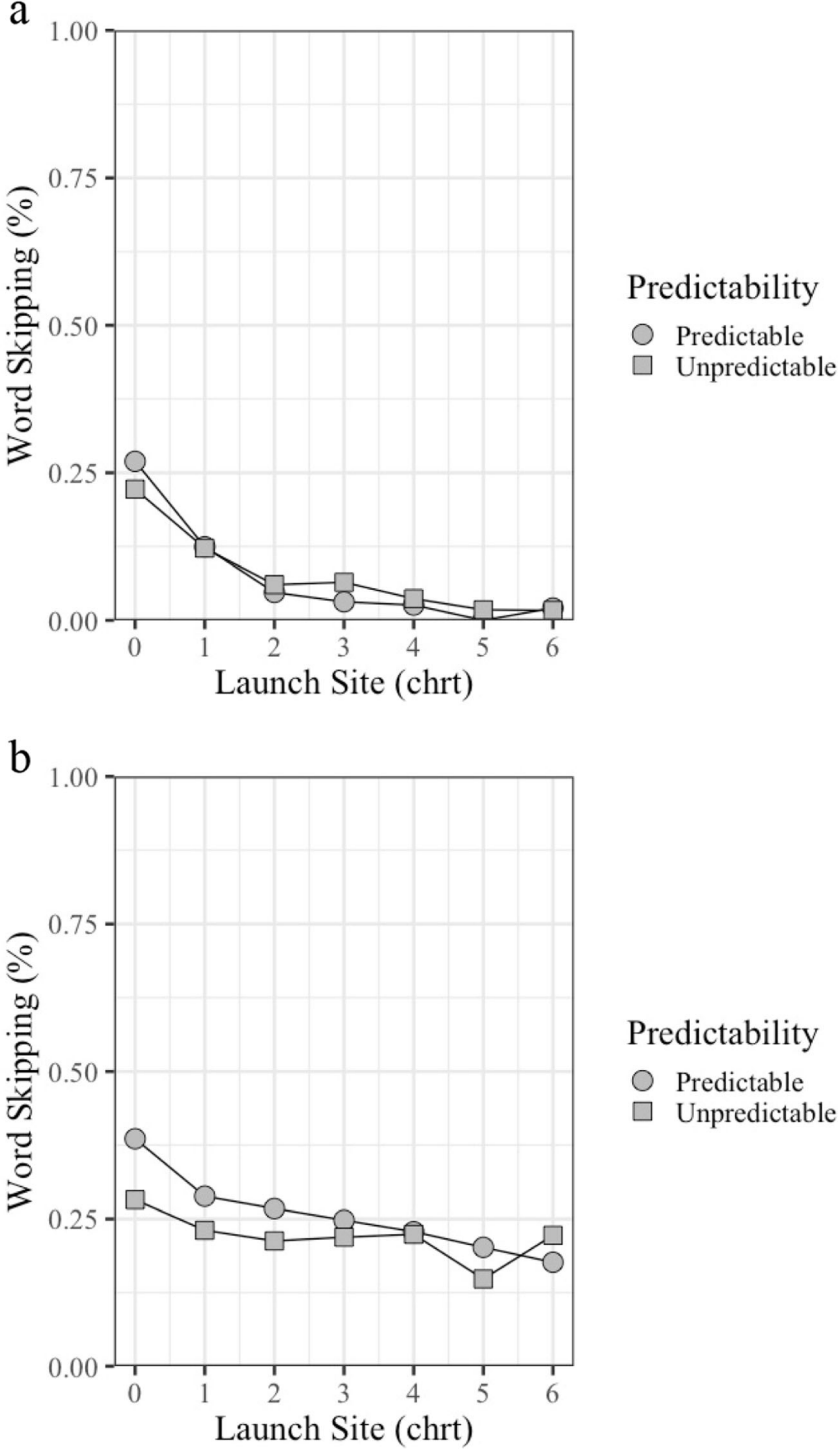
Effects of Saccade Launch Site on Word-Skipping Probability for (**a**) Experiment 1 and (**b**) Experiment 2

Mean target word-level measures are shown in Table 2 and statistical effects for hypothesis-testing variables are summarized in Table 3. No predictability effects were observed for word-skipping rates (|*z*| < 1), which were low for high- and low-predictability words (7.2% vs. 7.8%). We further explored possible effects of word predictability on target word-skipping, by including the launch site of the saccade as an additional variable. This is based on the observation that predictability effects on word-skipping are more likely to be observed when saccades are launched from locations closer to the target word (e.g., Fitzsimmons & Drieghe, 2011) see Fig. 3. Launch site was calculated as the distance (in degrees of visual angle) from the right border of the target word, which we converted into number of letters for transparency (based on average letter width in the stimulus set). Launch sites more than six letters from the target word were excluded, as readers were unlikely to obtain a useful preview of the target word from a fixation this distant (affecting 5.0% of saccades). With saccade launch site included as a continuous variable, there was no main effect of word predictability (*b* = 0.21, *SE* = 0.32, *z* = -0.65). While skipping rates were higher for launch sites closer to the target word (*b* = 0.82, *SE* = 0.08, *z* = -10.20), there was no interaction between word predictability and launch site (*b* = 0.20, *SE* = 0.16, *z* = 1.25). The indication, therefore, is that word-skipping was a low-frequency event that was not influenced by target-word predictability.

**Table 2:** Target word-level measures for Experiments 1 and 2

Eye-movement variables	Experiment 1		Experiment 2	
	High Predictability	Low Predictability	High Predictability	Low Predictability
Hypothesis-testing variables				
Word-skipping probability (%)	7.2 (1)	7.8 (1)	26.8 (1)	22.1 (1)
First-fixation duration (ms)	248 (2)	260 (2)	234 (2)	252 (3)
Gaze duration (ms)	287 (3)	305 (3)	255 (3)	281 (4)
Exploratory variables				
Single-fixation duration (ms)	258 (3)	277 (3)	240 (3)	263 (3)
Total reading time (ms)	322 (5)	378 (6)	289 (4)	350 (6)
Regressions-out (%)	5.4 (1)	6.6 (1)	8.0 (1)	8.5 (1)
Regressions-in (%)	3.3 (1)	9.2 (1)	4.8 (1)	12.4 (1)

*Note*. The standard error of the mean is shown in parentheses

**Table 3:** Word-level statistical effects for Experiments 1 and 2

Source		SKIP	FFD	GD
Experiment 1				
(Intercept)	Estimate	-3.12	255.48	297.61
	SE	0.24	5.44	7.37
	t/z	-12.83	46.97	40.41
Predictability	Estimate	0.11	12.66	18.42
	SE	0.26	4.10	5.93
	t/z	0.41	3.09*	3.10*
Experiment 2				
(Intercept)	Estimate	-1.17	243.90	262.52
	SE	0.14	4.61	5.71
	t/z	-8.36	52.93	46.83
Predictability	Estimate	-0.39	17.87	25.30
	SE	0.15	4.58	7.47
	t/z	-2.64*	3.91*	3.39*

*Denotes statistical significance (*t*/z > 2.39)

We observed clear effects of word predictability in both first fixations and gaze durations for target words that were not skipped (first-fixation duration, high-predictability, *M* = 248 ms, low-predictability, *M* = 260 ms; gaze duration, high-predictability, *M* = 287, low-predictability, *M* = 305 ms; |*t*|s > 3.08). Consistent with findings from Latin-based languages, such as English and German, fixation times were shorter when words were highly predictable, as compared to less predictable, from the prior sentence context.

### Exploratory word-level analyses

Additional word-level analyses indicated that target words had shorter single-fixation durations and total reading times when they were highly predictable rather than less predictable from the prior sentence context. In addition, there was a lower probability of a regression back to target words that were highly predictable rather than less predictable, with no indication of an effect of predictability on the probability of a regression from the target word.

The absence of a predictability effect in word-skipping is divergent from findings for Latin-based languages (Balota et al., 1985; Ehrlich & Rayner, 1981; Rayner & Well, 1996). This is a likely consequence of reduced word-skipping in Arabic reading, possibly due to difficulty identifying words parafoveally. Indeed, word-skipping effects emerged only for close launch sites that allow better parafoveal processing. In the *Introduction*, we proposed such effects might be attributable to specific characteristics of the Arabic script, including impoverished parafoveal processing of orthographic and morphological information, as well as the greater morphological complexity of Arabic words. While we do not systematically assess the contribution of these factors, Experiment 2 addressed this issue by examining effects for short words with a simple morphological structure. This was achieved by creating a new stimulus set (see Fig. 2) comprising only three- to four-letter target words that were morphologically simple (composed wholly or mostly of root consonants, without prefixes, such as the bigram , which preceded 80% of target words in Experiment 1). The crucial question was whether this would reveal a predictability effect in word-skipping.

## Experiment 2: Results and discussion

As in Experiment 1, comprehension accuracy was high (*M* = 97%) and above 90% for all participants.

### Sentence-level measures

Table 1 reports sentence-level means for Experiment 2. These were in line with eye-movement parameters in Experiment 1 and other Arabic studies (Hermena et al., 2017, 2019; Jordan et al., 2014; Paterson et al., 2015).

### Hypothesis-testing analyses for word-level measures

Mean target word-level measures are shown in Table 2 and statistical effects for variables used to test hypotheses concerning word predictability effects are summarized in Table 3. Word-skipping appeared to be higher in Experiment 2 than in Experiment 1 (Experiment 1, 7.5%; Experiment 2, 24.5%). Moreover, there was an effect of word predictability due to higher skipping of target words that were highly predictable (26.8%) rather than less predictable (22.1%, *z* = -2.64). As in Experiment 1, we explored this effect further by including saccade launch site as a continuous variable and excluding launch sites from beyond six letter spaces from the right edge of the target word (affecting 5.3% of saccades). This produced a main effect of word predictability (*b* = -0.57, *SE* = 0.23, *z* = -2.52), due to higher skipping probabilities for highly predictable compared to less predictable words. There was also a main effect of saccade launch site (*b* = -0.13, *SE* = 0.04, *z* = -3.94), due to higher skipping probabilities for launch sites closer to the target word. However, no significant interaction between these variables was observed (*b* = 0.07, *SE* = 0.07, *z* = 1.06).

As in Experiment 1, first-fixation durations and gaze durations for target words that were not skipped in first-pass reading yielded effects of word predictability, with shorter reading times for high- than low-predictability words (FFD = 234 ms vs. 252 ms, GD = 255 ms vs. 281 ms, |*t*|s > 3.38). Experiment 2 therefore revealed that predictability can influence both skipping probability and fixations times for words in Arabic reading, at least for words that are short and morphologically simple.

### Exploratory word-level analyses

Similar to Experiment 1, exploratory analyses of additional word-level measures suggested predictability effects in single-fixation durations and total reading times for words, with shorter reading times for highly predictable compared to less predictable words. As in Experiment 1, there was a higher probability of a regression back to highly predictable than less predictable target words, but no indication that predictability influenced the probability of a first-pass regression from these words.

## General discussion

We report two experiments investigating effects of word predictability in Arabic reading. Both show clear predictability effects in fixations times for words, consistent with findings for other scripts, including Latin-based scripts like English (e.g., Rayner & Well, 1996). However, a predictability effect on word-skipping for short, morphologically simple words in Experiment 2 was not observed for longer words with more varied structures in Experiment 1. Additional analyses that examined these effects as a function of saccade launch site (measured from up to six letter spaces to the right of the target word) showed no indication of a word predictability effect in Experiment 1, and no difference in the size of the word predictability effect as a function of launch site in Experiment 2. Our findings suggest that, while it is possible to observe predictability effects on word-skipping in Arabic reading, such events are likely to be rare, and possibly restricted to situations in which words are short, morphologically simple, and highly predictable from the prior sentence context.

This finding is in line with the more general observation that word-skipping occurs relatively infrequently in Arabic reading. In the present case, we observed low skipping rates, averaging about 7.5% for target words of variable length and complexity in Experiment 1, and around only 24.5% in Experiment 2 even though target words in this experiment were short and morphologically simple. We note also that the higher skipping rate in Experiment 2 relative to Experiment 1 could relate to differences in the spatial extent of target words across the two experiments. The average skipping rate for Experiment 1 was broadly similar to that reported in other Arabic studies (Hermena et al., 2017, 2019; Paterson et al., 2015). This contrasts with skipping rates for Latin-based scripts like English, which averages around 20–30%, rising to 60% or more for short words. How Arabic word-skipping compares with other Semitic languages, like Hebrew, currently is difficult to establish. One issue is that many Hebrew studies do not report skipping rates (e.g., Dank et al., 2015; Deutsch, 1998; Deutsch et al., 2000, 2003, 2005, 2021; Deutsch & Bentin, 2001; Kuperman & Deutsch, 2020; Nazir et al., 2004; Velan et al., 2013; Yablonski et al., 2017). However, data from one study, which examined word-length effects, showed quite high skipping rates, similar to that for Latin-based scripts, with approximately 30% skipping for short (three-letter) words, falling to about 18% for longer (seven-letter) words (Deutsch & Rayner, 1999). A further issue is that few studies have been conducted in other comparable languages. For example, it could be informative to assess word-skipping for Uyghur, which is an agglutinative Turkic language that uses the Arabic script but does not omit vowel information in words or use the Semitic morphology. However, such comparisons currently are difficult as relatively few studies have been reported for this script, and existing studies report variable skipping rates, ranging from about 8% in a study by Yan et al. (2014) to around 29% in a recent study by Zhou et al. (2021). It nevertheless seems clear from research to date that, as compared with readers of Latin-based languages like English, Arabic readers do not gain efficiency by skipping words, and fixate most words in a sentence at least once.

We argued in the *Introduction* that this low rate of word-skipping might be a consequence of specific orthographic and morphological characteristics of the Arabic script (see also AlJassmi et al., 2021). In particular, we noted that the use of a semi-cursive script with variable letter widths might limit the usefulness of parafoveal length information for constraining word identities. In addition, words might be more difficult to identify parafoveally because of close similarity in the shape of certain groups of letters (e.g., Eviatar et al., 2004). We also noted that readers might not benefit from parafoveal preview of beginning letters in words, as compared to in scripts like English, because Arabic uses a non-concatenative morphology in which the most informative elements (the consonant triple that conveys core word information) seldom appears at the word beginning (Farid & Grainger, 1996). Finally, the agglutinative (or fusional) nature of the Arabic morphology produces complex, informationally dense words that may be difficult to process parafoveally, and may also impose foveal processing costs for fixated words that limit resources for parafoveal processing (e.g., Henderson & Ferreira, 1990; Payne et al., 2016; Roman & Pavard, 1987). Such costs may explain why word-skipping is infrequent and why re-fixation probabilities (whereby words receive multiple fixations) are high (e.g., Paterson et al., 2015).

This raises the question of what information can be processed parafoveally in Arabic reading. This question has received relatively limited attention to date, although there is evidence that readers are sensitive to the use of diacritics to disambiguate words (Hermena et al., 2016). These are glyph-like marks placed above or below Arabic letters to convey vowel information. Diacritics are seldom used unless required to disambiguate words (but are always present in religious texts and widely used in texts designed for beginning readers). The study by Hermana et al. used the boundary paradigm (Rayner, 1975) to make surreptitious changes to parafoveal previews of diacritics ahead of the reader fixating a specific target word in each sentence. When this information was inaccurate, it disrupted subsequent processing of the target word, suggesting that this information was available during parafoveal processing. This finding therefore reveals sensitivity to subtle orthographic cues during parafoveal processing. Moreover, it establishes the boundary paradigm as a method for investigating other aspects of parafoveal orthographic processing in Arabic reading, including the use of cues to word length and the precision with which letter identities can be processed. Moreover, the technique has the capacity to reveal how contextual constraints and visual and orthographic factors act together to guide eye movements (e.g., Balota et al., 1985; Choi et al., 2017; White et al., 2005).

Similar considerations may apply to morphological factors. Evidence from research in Hebrew suggests readers can acquire quite detailed morphological information during parafoveal processing. For example, several studies using the boundary paradigm show that readers are sensitive to the presence of letter sequences that convey the core meaning of a word (Deutsch et al., 2003, 2005). In Hebrew, as in Arabic and other Semitic languages, words are constructed using a non-concatenative derivational morphology in which a sequence of letters that form the word’s root, which captures its core meaning, interleave with other letters in a word (see Deutsch et al., 1998). Studies using the boundary paradigm show that preview of a word containing the same root as a target word, as compared with other shared letters, results in shorter target word fixations (Deutsch et al., 2003), and that the use of morphological preview information can be mediated by context (Deutsch et al., 2005). The indication, therefore, is that detailed morphological information can be acquired parafoveally during Hebrew reading. This raises the question of whether similar information is obtained in Arabic reading. While evidence exists to suggest this might be the case (e.g., Hermena et al., 2019; Hermena et al., 2021), this issue requires further investigation, including needing to establish whether parafoveal processing of morphological information is influenced by contextual constraints. Moreover, this approach might also be used to investigate whether the location of root information can influence processing, such that parafoveal preview effects might be stronger when this information is closer to the beginning of words.

The present findings might also be considered in the context of a debate concerning whether lexical prediction can be observed in the absence of parafoveal information. We argued in the *Introduction* that the decision to skip a word is based on both predictions derived from prior context and parafoveal preview information. This is in line with the view that at least partial parafoveal information is required to support lexical prediction (seeStaub, 2015 ; Staub & Goddard, 2019). Evidence for this view comes from studies using the boundary paradigm to compare effects of valid and invalid previews. In these experiments, an invisible boundary is placed in front of a target word in a sentence. Before the reader’s gaze crosses this boundary, the target word is shown as a normal (i.e., a valid) preview or masked (e.g., by replacing the target word’s letters with either visually similar or dissimilar letters) to create an invalid preview. As soon as the reader’s gaze crosses the boundary, the target word quickly reverts to normal, allowing the experimenter to compare effects of valid and invalid previews on subsequent processing. Crucially, studies using this approach show that predictability effects are obtained only following valid previews or visually similar invalid previews (e.g., Balota et al., 1985; Staub & Goddard, 2019; also Chang et al., 2020a,b).

This observation has led researchers to argue that lexical prediction facilitates the early processing of a word by pre-activating its features and letters, and that this depends crucially on the availability of parafoveal information (Staub, 2015; Staub & Goddard, 2019). However, an alternative account holds that readers can use lexical prediction to guess upcoming words even in the absence of parafoveal information, and that effects in the boundary paradigm may arise from specific processing costs for invalid previews. Support for this alternative view comes from research showing that predictability effects can be obtained even for words appears at the beginning of a line of text, and therefore in the absence of parafoveal preview (Parker et al., 2017). We have argued that parafoveal processing of Arabic words frequently is impoverished because of specific orthographic and morphological characteristics of the script. Moreover, we showed in Experiment 1, using words varying in length and morphological complexity, that this can eliminate predictability effects in word-skipping. Might these results therefore provide further evidence that lexical prediction can take place in the absence of parafoveal information? We think not as we observed clear predictability effects in fixation times for words. What instead seems likely is that the typically more impoverished parafoveal processing of words in Arabic reading limits the influence of lexical prediction so that it seldom affects word-skipping.

This raises the question of how the present findings might be accommodated by models of eye-movement control. The currently dominant E-Z Reader and SWIFT models were designed to simulate eye movement for Latin-based languages like English and German. It is therefore important to establish the generality of these models by considering their capacity to explain effects in other scripts, including Arabic. The models can accurately simulate effects of key lexical variables (word length, frequency and predictability) on the probability and duration of fixations on words in English and German (e.g., Engbert et al., 2005; Reichle, 2021; Reichle et al., 1998). It seems likely that the models can also account for word length and frequency effects in Arabic reading (Hermena et al., 2017, 2019; Paterson et al., 2015), as well as the word predictability effects in the present study. The E-Z Reader models might account for predictability effects on fixation times by assuming that this influences the familiarity check (i.e., L1 processing) for these words. The model might also account for the limited influence of predictability on word-skipping by assuming that a familiarity check for parafoveal words proceeds more quickly for shorter words that are morphologically simple, such as the target words in Experiment 2 rather than the more varied words used in Experiment 1. In a similar vein, SWIFT might accommodate word predictability effects in fixation times for both types of words, and allow for skipping of the shorter and morphologically simpler target words in Experiment 2 to be more affected by contextual constraints, as compared to the more varied items in Experiment 1.

Beyond these findings, it should be clear that we currently lack a model of eye-movement control that can account for more specific influences of Arabic orthography and morphology (see Hermena & Reichle, 2020, for further discussion). This necessarily will include mechanisms that can account for effects of the locations of core (i.e., root) information and number of morphological units on the processing of words (AlJassmi et al., 2021; Tibi et al., 2020; Tibi & Kirby, 2017). Consequently, while existing models might account for the lexical effects reported in the present experiments, more sophisticated mechanisms will be required to account for the influence of Arabic orthography and morphology on eye-movement control in reading.
